# Supplementary material for: An Observational Case-Control Study on Parental Age and Childhood Renal Tumors
Source: Cancers (Basel). 2023 Oct 26;15(21):5144. doi: 10.3390/cancers15215144 (PMC10647705; doi:10.3390/cancers15215144)
Supplement: Supplementary file 1 [file cancers-15-05144-s001.zip › cancers-2670025-supplementary.pdf]

# Supplementary Materials: An Observational Case Control Study on Parental Age and Childhood Renal Tumors

**Georgios Politis, Stefan Wagenpfeil, Nils Welter, Marvin Mergen, Rhoikos Furtwängler and Norbert Graf**

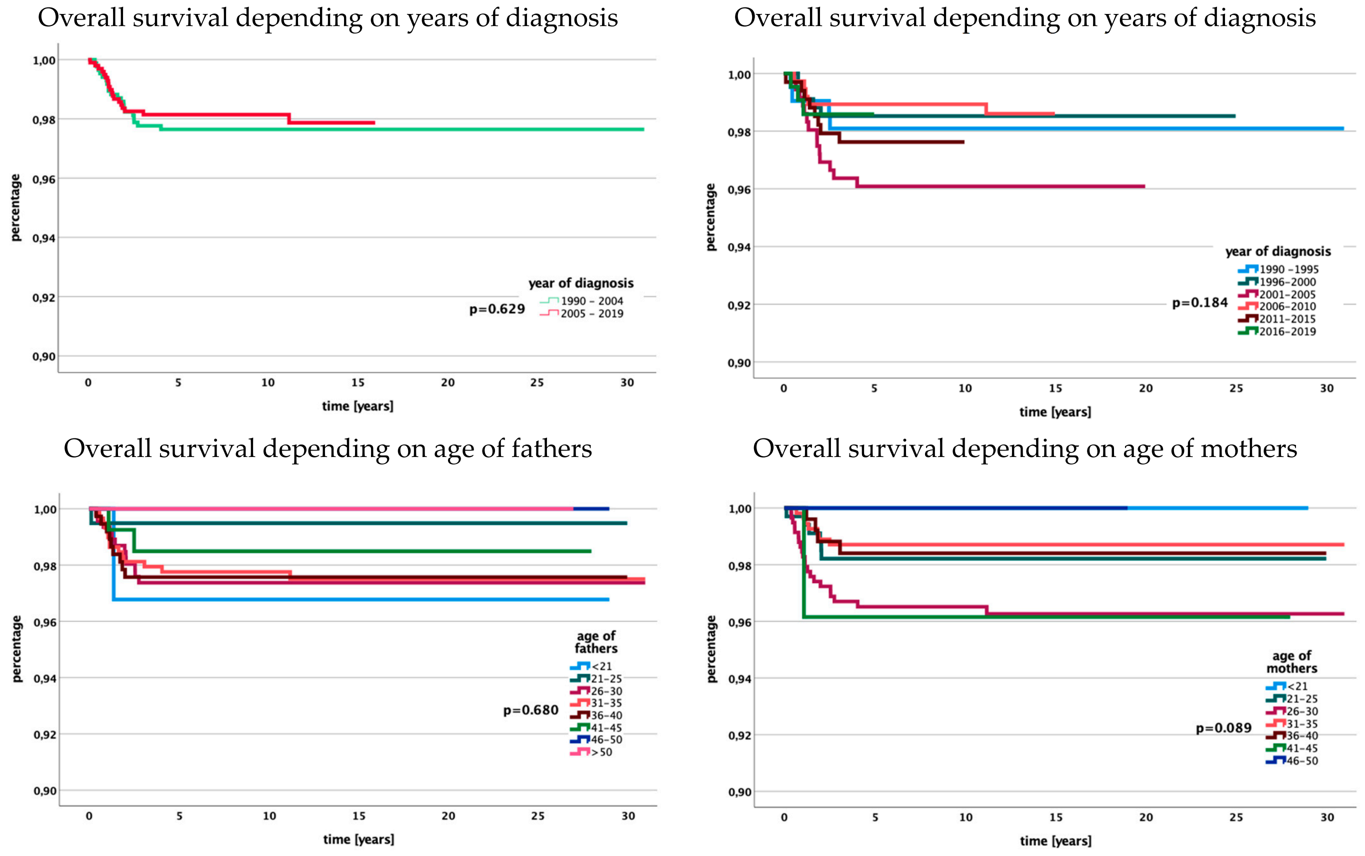


**Figure S1.** Kaplan-Meier analysis for **o**verall survival of patients with a nephroblastoma depending on the year of diagnosis or the age of fathers or mothers.

**Table S1.** Characteristics of patients and their parents. (WT: Wilms tumor / nephroblastoma).

| | | | |
|---|---|---|---|
| **patients** | **number of patients** | 2277 | |
| | **gender** | | |
| | male | 1080 | 47.4% |
| | female | 1197 | 52.6% |
| | **mean age [years]** | 3.37 | ± 2.81 |
| | **tumor localization** | | |
| | right | 1013 | 44.5% |
| | left | 1052 | 46.2% |
| | bilateral | 197 | 8.7% |
| | extrarenal | 15 | 0.6% |
| | **histology** | | |
| | WT | 1923 | 84.5% |
| | *low risk* | *78* | *4.1% of WT* |
| | *intermediate risk* | *1624* | *84.5% of WT* |
| | *high risk* | *221* | *11.4% of WT* |

| | | | |
|---|---|---|---|
| | non-Wilms tumor | 354 | 15.5% |
| | **metastasis** | 347 | 15.2% |
| | **local stage** | | |
| | I | 1262 | 55.4% |
| | II | 470 | 20.6% |
| | III | 416 | 18.3% |
| | missing | 129 | 5.7% |
| | **relapse** | 274 | 12.0% |
| | **died** | 63 | 2.8% |

| | | | |
|---|---|---|---|
| **parents** | **mean age of fathers at the time of diagnosis [years]** | 32.77 | ± 6.39 |
| | **mean age of mothers at the time of diagnosis [years]** | 29.68 | ± 5.39 |
| | **mean age difference between fathers and mothers [years]** | 2.96 | ± 4.77 |

**Table 2.** Cox regression analysis for overall survival.

| | Hazard ratio | 95% confidence interval for Hazard ratio | p-value |
|---|---|---|---|
| **year of diagnosis** | 1.532 | 0.702 – 3.343 | 0.284 |
| **age of the father at the time of birth of the child** | 0.963 | 0.890 – 1.042 | 0.346 |
| **age of the mother at the time of birth of the child** | 1.068 | 0.982 – 1.162 | 0.122 |
| **year of child's birth** | 0.641 | 0.294 – 1.397 | 0.264 |
| **age of the child with kidney cancer** | 0.434 | 0.729 – 0.331 | 0.434 |
| **histology** | | | < 0.001 |
| intermediate risk | 1.305 | 0.175 – 9.734 | 0.029 |
| high risk | 4.910 | 0.647 – 37.240 | < 0.001 |
| **local stage** | | | < 0.001 |
| II | 3.483 | 1.132 – 10,713 | 0.029 |
| III | 13.098 | 4.951 – 36.647 | < 0.001 |
